# ProtTrans-Glutar: Incorporating Features From Pre-trained Transformer-Based Models for Predicting Glutarylation Sites

**DOI:** 10.3389/fgene.2022.885929

**Published:** 2022-05-31

**Authors:** Fatma Indriani, Kunti Robiatul Mahmudah, Bedy Purnama, Kenji Satou

**Affiliations:** ^1^ Graduate School of Natural Science and Technology, Kanazawa University, Kanazawa, Japan; ^2^ Department of Computer Science, Lambung Mangkurat University, Banjarmasin, Indonesia; ^3^ Department of Postgraduate of Mathematics Education, Universitas Ahmad Dahlan, Yogyakarta, Indonesia; ^4^ School of Computing, Telkom University, Bandung, Indonesia; ^5^ Institute of Science and Engineering, Kanazawa University, Kanazawa, Japan

**Keywords:** lysine glutarylation, protein sequence, transformer-based models, protein embedding, machine learning, binary classification, imbalanced data classification, post-translation modification

## Abstract

Lysine glutarylation is a post-translational modification (PTM) that plays a regulatory role in various physiological and biological processes. Identifying glutarylated peptides using proteomic techniques is expensive and time-consuming. Therefore, developing computational models and predictors can prove useful for rapid identification of glutarylation. In this study, we propose a model called ProtTrans-Glutar to classify a protein sequence into positive or negative glutarylation site by combining traditional sequence-based features with features derived from a pre-trained transformer-based protein model. The features of the model were constructed by combining several feature sets, namely the distribution feature (from composition/transition/distribution encoding), enhanced amino acid composition (EAAC), and features derived from the ProtT5-XL-UniRef50 model. Combined with random under-sampling and XGBoost classification method, our model obtained recall, specificity, and AUC scores of 0.7864, 0.6286, and 0.7075 respectively on an independent test set. The recall and AUC scores were notably higher than those of the previous glutarylation prediction models using the same dataset. This high recall score suggests that our method has the potential to identify new glutarylation sites and facilitate further research on the glutarylation process.

## 1 Introduction

Similar to the epigenetic modification of histones and nucleic acids, the post-translational modification (PTM) of amino acids dynamically changes the function of proteins and is actively studied in the field of molecular biology. Among various kinds of PTMs, lysine glutarylation is defined as an attachment of a glutaryl group to a lysine residue of a protein (Lee et al., 2014). This modification was first detected *via* immunoblotting and mass spectrometry analysis and later validated using chemical and biochemical methods. It is suggested that this PTM may be a biomarker of aging and cellular stress (Harmel and Fiedler, 2018). Dysregulation of glutarylation is related to some metabolic diseases, including type 1 glutaric aciduria, diabetes, cancer, and neurodegenerative diseases (Tan et al., 2014; Osborne et al., 2016; Carrico et al., 2018). Since the identification of glutarylated peptides using proteomics techniques is expensive and time-consuming, it is important to investigate computational models and predictors to rapidly identify glutarylation.

Based on a survey of previous research, various prediction models have been proposed to distinguish glutarylation sites. The earliest one, GlutPred (Ju and He, 2018), constructs features from amino acid factors (AAF), binary encoding (BE), and the composition of k-spaced amino acid pairs (CKSAAP). The authors selected 300 features using the mRMR method. To overcome the problem of imbalance in this dataset, a biased version of support vector machine (SVM) was employed to build the prediction model. Another predictor, iGlu-Lys (Xu et al., 2018), investigated four different feature sets, physicochemical properties (AAIndex), K-Space, Position-Special Amino Acid Propensity (PSAAP), and Position-Specific Propensity Matrix (PSPM), in conjunction with SVM classifier. The feature set PSPM performed best in the 10-fold cross-validation and was therefore applied to the model. iGlu-Lys performed better than GlutPred in terms of accuracy and specificity scores. However, their sensitivity scores were lower. The next model proposed, MDDGlutar (Huang et al., 2019), divided the training set into six subsets using maximal dependence decomposition (MDD). Three feature sets were evaluated separately using SVM: amino acid composition (AAC), amino acid pair composition (AAPC), and CKSAAP. The best cross-validation score was the AAC feature set. The results of independent testing yielded a balanced score of 65.2% sensitivity and 79.3% specificity, but it had lower specificity and accuracy than those of the GlutPred model.

The next two predictors included the addition of new glutarylated proteins from *Escherichia coli* and HeLa cells for their training and test sets. RF-GlutarySite (Al-barakati et al., 2019) utilizes features constructed from 14 feature sets, reduced with XGBoost. The model’s reported performance for independent testing was balanced, with 71.3% accuracy, 74.1% sensitivity, and 68.5% specificity. However, it is interesting to note that the test data was balanced by under-sampling, which did not represent a real-world scenario. iGlu_Adaboost (Dou et al., 2021) sought to fill this gap by using test data with no resampling. This model utilizes features from 188D, enhanced amino acid composition (EAAC), and CKSAAP. With the help of Chi2 feature selection, 37 features were selected to build the model using SMOTE-Tomek re-sampling and the Adaboost classifier. The test result had good performance for recall, specificity, and accuracy metrics, but a lower Area Under the Curve (AUC) score than that of previous models.

Although many models have been built to distinguish between positive and negative glutarylation sites, the performance of these methods remains limited. One challenge to this problem is finding a set of features to represent the protein subsequence, which enables a correct classification of glutarylation site. BERT models (Devlin et al., 2019), and other transformer-based language models from natural language processing (NLP) research, show excellent performance for NLP tasks. These language models, having been adapted to biological sequences by treating them as sentences and then trained using large-scale protein corpora (Elnaggar et al., 2021), also show promise for various machine learning tasks in the bioinformatics domain.

Previous studies have investigated the use of pre-trained language models from BERT and BERT-like models to show its effectiveness as protein sequence representation for protein classification. For example, Ho et al. (2021) proposed a new approach to predict flavin adenine dinucleotide (FAD) binding sites from transport proteins based on pre-training BERT, position-specific scoring matrix profiles (PSSM), and an amino acid index database (AAIndex). Their approach showed an accuracy score of 85.14%, which is an improvement over the scores of the previous methods. Another study (Shah et al., 2021) extracted features using pre-trained BERT models to discriminate between three families of glucose transporters. This method, compared to two well-known feature extraction methods, AAC and DPC, showed an improved performance of more than 4% in average sensitivity and Matthews correlation coefficient (MCC). In another study, Liu built a predictor for protein lysine glycation sites using features extracted from pre-trained BERT models, which showed improved performance in terms of accuracy and AUC score compared to previous methods (Liu et al., 2022). These studies demonstrate the suitability of utilizing BERT models to improve various protein classification tasks. Therefore, using embeddings from pre-trained BERT and BERT-like models has the potential to build an improved glutarylation prediction model.

**FIGURE 1:**
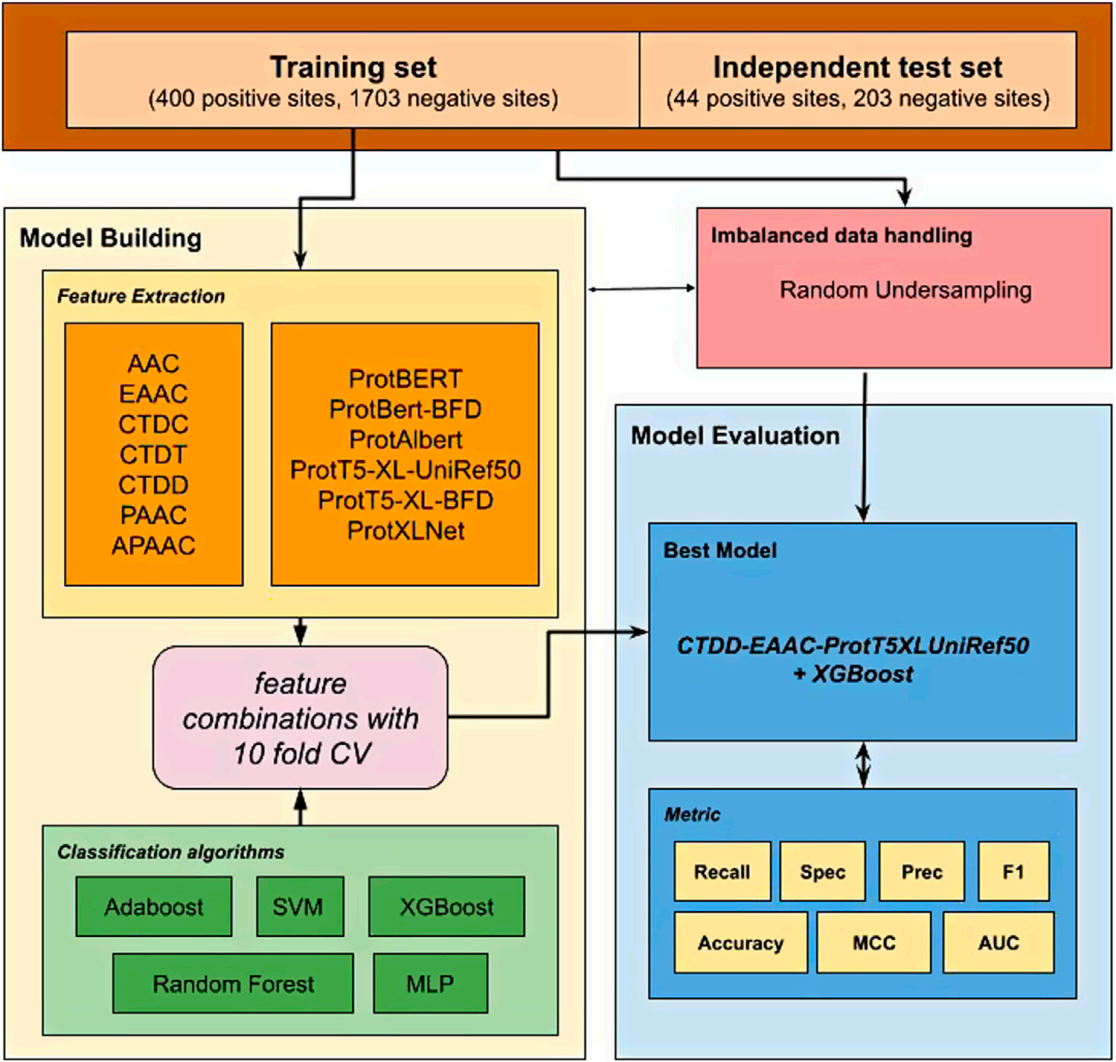
Workflow strategy for the development of ProTrans-Glutar model.

In this study, we proposed a new prediction model to predict glutarylation sites (Figure 1) by incorporating features extracted from pre-trained protein models combined with features from handcrafted sequence-based features. A public dataset provided from Al-barakati et al. (2019) was used in this study. It was an imbalanced dataset with 444 positive sites and 1906 negative sites, and already separated into two sets for use in model building and independent testing. First, various feature sets were extracted from the dataset, consisting of two types of features. The first type consists of seven classic sequence-based features, and the second type consists of six embeddings from pre-trained protein language models. We evaluated the classifiers using a 10-fold cross-validation for the individual feature set. The next step was to combine two or more feature sets to evaluate further models, such as AAC-EAAC, AAC-CTDC, and AAC-ProtBert. For this, we limited the embedding features to a maximum of one in the combination. Five classification algorithms were included in the experiments: Adaboost, XGBoost, SVM (with RBF kernel), random forest (RF), and multilayer perceptron (MLP). Our best model combines the features of CTDD, AAC, and ProtT5-XL-UniRef50 with the XGBoost classification algorithm. This model, with the model of the best feature set from sequence-based feature groups and the model of the best feature set from the protein embedding feature group, was then evaluated with an independent dataset. For independent testing, the entire training set was used to develop a model. In both model building and independent testing, a random under-sampling method was used to balance the training dataset, while the testing dataset was not resampled to reflect performance in the real-world unbalanced scenario.

## 2 Materials and Methods

### 2.1 Dataset

This study utilized unbalanced benchmark datasets compiled by Al-barakati et al. (2019) to build their predictor, RF-GlutarySite. This dataset collected positive glutarylation sites from various sources, including PLMD (Xu et al., 2017) and (Tan et al., 2014) and consisted of four different species (*Mus musculus*, *Mycobacterium tuberculosis*, *E. coli*, and HeLa cells), for a total of 749 sites from 234 proteins. Homologous sequences that showed ≥40% sequence identity were removed using the CD-HIT tool. The remaining proteins were converted into peptides with a fixed length of 23, with glutarylated lysine as the central residue, and 11 residues each upstream and downstream. Negative sites were generated in the same way, but the central lysine residue was not glutarylated. After removing homologous sequences, the final dataset consisted of 453 positive and 2043 negative sites. The distributions of the training and testing datasets are listed in Table 1. This dataset was also used by Dou et al. (2021) to build the proposed predictor model iGlu_Adaboost (Dou et al., 2021).

**TABLE 1:** Number of positive and negative sites in training and test set.

	Training set	Test set	
Positive sites	400	44	444
Negative sites	1703	203	1906
	2103	247	

### 2.2 Feature Extraction

The extraction of numerical features from protein sequences or peptides is an important step before they can be utilized by machine learning algorithms. In this study, we investigated two types of features: classic sequence-based features and features derived from pre-trained transformer-based protein embeddings. Classic sequence-based features were extracted using the *iFeature* Python package (Chen et al., 2018). After preliminary experiments, seven feature groups were chosen for further investigation: AAC, EAAC, Composition/Transition/Distribution (CTD), pseudo-amino acid composition (PAAC), and amphiphilic pseudo-amino acid composition (APAAC). The second type of feature, embeddings from pre-trained transformer-based models, was extracted using models trained and provided by Elnaggar et al. (2021). It consists of six feature sets from six protein models: ProtBERT, ProtBert-BFD, ProtAlbert, ProtT5-XL-UniRef50, ProtT5-XL-BFD, and ProtXLNet. The data for all extracted features are provided in the Supplementary Material.

#### 2.2.1 Amino Acid Composition and Enhanced Amino Acid Composition

The AAC method encodes a protein sequence-based on the frequency of each amino acid (Bhasin and Raghava, 2004). For this type of feature, we used two variants.

The first variant is the basic AAC, in which the protein sequence is converted into a vector of length 20, representing the frequency of the 20 amino acids (“*ACDEFGHIKLMNPQRSTVWY*”). Each element is calculated according to Eq. 1, as follows:

$$f\left(t\right)=\frac{N\left(t\right)}{N}$$ (1)

where *t* is the amino acid type, *N(t)* is the total number of amino acids *t* appearing in the sequence, and *N* is the length of the sequence.

The second variant is EAAC, introduced by Chen et al. (2018). In this encoding, the EAAC was calculated using sliding windows, that is, from a fixed window size, moving from left to right. To calculate the frequency of each amino acid in each window, see Eq. 2:

$$f\left(t,win\right)=\frac{N\left(t,win\right)}{N\left(win\right)}$$ (2)

where *N*(*t,win*) represents the number of amino acids *t* that appear in the window *win* and *N*(*win*) represents the length of the window. To develop our model, a default window size of five was used. How these methods are applied to a protein sequence are provided in Supplementary File S1.

#### 2.2.2 Composition/Transition/Distribution

The CTD method encodes a protein sequence-based on various structural and physicochemical properties (Dubchak et al., 1995; Cai, 2003). Thirteen properties were used to build the features. Each property was divided into three groups (see Table 2). For example, the attribute “Hydrophobicity_PRAM900101” divides the amino acids into polar, neutral, and hydrophobic groups.

**TABLE 2:** Physicochemical attributes and its division of the amino acids.

Attribute		Division	
Hydrophobicity_PRAM900101	Polar: RKEDQN	Neutral: GASTPHY	Hydrophobicity: CLVIMFW
Hydrophobicity_ARGP820101	Polar: QSTNGDE	Neutral: RAHCKMV	Hydrophobicity: LYPFIW
Hydrophobicity_ZIMJ680101	Polar: QNGSWTDERA	Neutral: HMCKV	Hydrophobicity: LPFYI
Hydrophobicity_PONP930101	Polar: KPDESNQT	Neutral: GRHA	Hydrophobicity: YMFWLCVI
Hydrophobicity_CASG920101	Polar: KDEQPSRNTG	Neutral: AHYMLV	Hydrophobicity: FIWC
Hydrophobicity_ENGD860101	Polar: RDKENQHYP	Neutral:SGTAW	Hydrophobicity: CVLIMF
Hydrophobicity_FASG890101	Polar: KERSQD	Neutral: NTPG	Hydrophobicity: AYHWVMFLIC
Normalized van der Waals volume	Volume range: 0–2.78	Volume range: 2.95–94.0	Volume range: 4.03–8.08
	GASTPD	NVEQIL	MHKFRYW
Polarity	Polarity value: 4.9–6.2	Polarity value: 8.0–9.2	Polarity value: 10.4–13.0
	LIFWCMVY	PATGS	HQRKNED
Polarizability	Polarizability value: 0–1.08	Polarizability value: 0.128–120.186	Polarizability value: 0.219–0.409
	GASDT	GPNVEQIL	KMHFRYW
Charge	Positive: KR	Neutral: ANCQGHILMFPSTWYV	Negative: DE
Secondary structure	Helix: EALMQKRH	Strand: VIYCWFT	Coil: GNPSD
Solvent accessibility	Buried: ALFCGIVW	Exposed: PKQEND	Intermediate: MPSTHY

The CTD feature comprises three parts: composition (CTDC), transition (CTDT), and distribution (CTDD). For composition, an attribute contributes to three values, representing the global distribution (frequency) of the amino acids in each of the three groups of attributes. The composition is computed as follows:

$$C\left(r\right)=\frac{N\left(r\right)}{N}$$ (3)

where *N*(*r*) is the number of occurrences of type *r* amino acids in the sequence and *N* is the length of the sequence.

For transition, an attribute also contributes to three values, each representing the number of transitions between any pair of groups. The transition is calculated as follows:

$$T\left(r,s\right)=\frac{N\left(r,s\right)+N\left(s,r\right)}{N-1}$$ (4)

where *N*(*r,s*) represents the number of occurrences amino acid type *r* transit to type *s* (i.e., it appeared as “rs” in the sequence), and *N* is the length of the sequence. Similarly, *N*(*s,r*) is the reverse, that is, the number of “sr” occurrences in the sequence.

The distribution feature consists of five values per attribute group, each of which corresponds to the fraction of the sequence length at five different positions in the group: first occurrence, 25%, 50%, 75%, and 100%.

#### 2.2.3 Pseudo Amino Acid Composition

Pseudo amino acid composition feature was proposed by Chou (2001). For protein sequence P with L amino acid residues P = (R_1_R_2_R_3_…R_L_), the PAAC features can be formulated as

$$P={\left[p_{1},p_{2},\ldots ,p_{20},p_{20+1},\ldots ,p_{20+\lambda }\right]}^{T},\left(\lambda <L\right)$$ (5)

where

$$p_{u}=\{\begin{array}{c} \frac{f_{u}}{\sum_{i=1}^{20}f_{i}+w\sum_{k=1}^{\lambda }\tau_{k}},\left(1\leq u\leq 20\right)\\ \frac{w\tau_{u-20}}{\sum_{i=1}^{20}f_{i}+w\sum_{k=1}^{\lambda }\tau_{k}},\left(20+1\leq u\leq 20+\lambda \right) \end{array}$$ (6)

*w* is the weight factor and $\tau_{k}$ is the k-the tier correlation factor, defined as

$$\tau_{k}=\frac{1}{L-k}\sum_{i=1}^{L-K}J_{i,i+k},\;\left(k<L\right)$$ (7)

and

$$J_{i,i+k}=\frac{1}{\Gamma }\sum_{q=1}^{\Gamma }{\left[\Phi_{q}R_{i+k}-\Phi_{q}R_{i}\right]}^{2}$$ (8)

where Ф_*q*_(*R*_*i*_) is the *q*-th function of the amino acid *R*_*i*_, and Г the total number of functions. In here Г = 3 and the functions used are hydrophobicity value, hydrophilicity value, and side chain mass of amino acid *R*_*i*_.

A variant of PAAC called amphiphilic pseudo amino acid composition (APAAC) proposed in Chou (2005). A protein sample P with L amino acid residues P = (R_1_R_2_R_3_…R_L_), is formulated as

$$P={\left[p_{1},p_{2},\ldots ,p_{20},p_{20+1},\ldots ,p_{20+\lambda },p_{20+\lambda },\ldots ,p_{2\lambda }\right]}^{T},\left(\lambda <L\right)$$ (9)

where

$$p_{u}=\{\begin{array}{c} \frac{f_{u}}{\sum_{i=1}^{20}f_{i}+w\sum_{j=1}^{2\lambda }\tau_{j}},\left(1\leq u\leq 20\right)\\ \frac{w\tau_{u-20}}{\sum_{i=1}^{20}f_{i}+w\sum_{j=1}^{2\lambda }\tau_{j}},\left(20+1\leq u\leq 20+2\lambda \right) \end{array}$$ (10)

τ_j_ is the j-tier sequence-correlation factor calculated using the equations:

$$\{\begin{array}{c} \tau_{1}=\frac{1}{L-1}\sum_{i=1}^{L-1}H_{i,i+1}^{1}\\ \tau_{2}=\frac{1}{L-1}\sum_{i=1}^{L-1}H_{i,i+1}^{2}\\ \tau_{3}=\frac{1}{L-2}\sum_{i=1}^{L-2}H_{i,i+2}^{1}\\ \tau_{4}=\frac{1}{L-2}\sum_{i=1}^{L-2}H_{i,i+2}^{2},\lambda <L\\ \cdots \\ \tau_{2\lambda -1}=\frac{1}{L-1}\sum_{i=1}^{L-\lambda }H_{i,i+\lambda }^{1}\\ \tau_{2\lambda }=\frac{1}{L-1}\sum_{i=1}^{L-\lambda }H_{i,i+\lambda }^{1} \end{array}$$ (11)

where *H*_*i,j*_^1^ and *H*_*i,j*_^2^ are hydrophobicity and hydrophilicity values of the *i*-th amino acid, described by the following equation:

$$H_{i,j}^{1}=h^{1}\left(R_{i}\right)\cdot h^{1}\left(R_{j}\right)$$

$$H_{i,j}^{2}=h^{2}\left(R_{i}\right)\cdot h^{2}\left(R_{j}\right)$$ (12)

#### 2.2.4 Pre-Trained Transformer Protein Embeddings

Protein language models has been trained from large protein corpora, using the state-of-the-art transformer models from the latest NLP research (Elnaggar et al., 2021). Six of the models were applied to extract features for our task of predicting glutarylation sites.

• ProtBERT and ProtBert-BFD are derived from the BERT model (Devlin et al., 2019), trained on UniRef100 and BFD corpora, respectively.
• ProtT5-XL-UniRef50 and ProtT5-XL-BFD are derived from the T5 model (Raffel et al., 2020), trained on UniRef50 and BFD corpora, respectively.
• ProtAlbert is derived from the Albert model (Lan et al., 2020) trained on UniRef100 corpora.
• ProtXLNet is derived from the XLNet model (Yang et al., 2020), trained on UniRef100 corpora.

Protein embeddings (features) were extracted from the last layer of this protein language model to be used for subsequent supervised training. This layer is a 2-dimensional array with a size of 1024 × length of sequence, except for the ProtAlbert model with an array size of 4096 × length of sequence. For the glutarylation prediction problem, this feature is simplified by summing the vectors along the length of the sequence; hence, each feature group is now one-dimensional, with a length of 4,096 for ProtAlbert and 1,024 for the rest.

#### 2.2.5 The Feature Space

The features collected were of different lengths, as summarized in Table 3. These feature groups are evaluated either individually or using various combinations of two or more feature groups. As an example, for the combined feature group AAC-EAAC, a training sample will have 20 + 380 = 400-dimensional features.

**TABLE 3:** Features investigated for method development.

Group	Feature set	Length of features
Amino acid composition	AAC	20
	EAAC	380
C/T/D	CTDC	39
	CTDT	39
	CTDD	195
Pseudo amino acid composition	PAAC	35
	APAAC	50
Embeddings from pretrained transformer-based model	ProtBERT	1,024
	ProtBert-BFD	1,024
	ProtAlbert	4,096
	ProtT5-XL-UniRef50	1,024
	ProtT5-XL-BFD	1,024
	ProtXLNet	1,024

### 2.3 Imbalanced Data Handling

A class imbalance occurs when the number of samples is unevenly distributed. The class with a higher number of samples is called the majority class or the negative class, whereas the class with a smaller number is called the minority class. In the glutarylation dataset, the number of negative samples was nearly four times that of positive samples. This imbalance may affect the performance of classifiers because they are more likely to predict a positive sample as a negative sample (He and Garcia, 2009). A common strategy to solve this problem is by data re-sampling, either adding minority samples (over-sampling) or reducing majority samples (under-sampling). In this study, we implemented a random under-sampling strategy (He and Ma, 2013) after preliminary experiments with various re-sampling methods.

### 2.4 Machine Learning Methods

In this study, we used the XGBoost classifier (Chen and Guestrin, 2016) from the XGBoost package on the Python language platform (https://xgboost.ai). This is an implementation of a gradient-boosted tree classifier (Friedman, 2001). Gradient-boosted trees are an ensemble classifier built from multiple decision trees, constructed one by one. XGBoost has been successfully used in various classification tasks, including bioinformatics (Mahmud et al., 2019; Chien et al., 2020; Zhang et al., 2020). In our experiments, several other popular classifiers are also compared and evaluated, including SVM, RF, MLP, and Adaboost, provided by the scikit-learn package (https://scikit-learn.org).

### 2.5 Model Evaluation

To achieve the model with the best prediction performance, the model was evaluated using 10-fold cross-validation and an independent test. For cross-validation, the training dataset was randomly split into 10 folds of nearly equal size. Nine folds were combined and then randomly under-sampled for training, and the 10th fold was used for evaluation. This process was performed with the other combination of folds (nine for training and one for testing). To remove sampling bias, the cross-validation process was repeated three times, and the mean performance was reported as the CV result. For independent testing, the entire training data were randomly under-sampled, then used to build the model, and later evaluated using the independent test set. Since the randomness in the under-sampling may affect to the performance result, this testing was repeated five times, and the mean performance was reported as an independent test result.

The performance of the cross-validation and independent test results was evaluated using seven performance metrics: recall (Rec), specificity (Spe), precision (Pre), accuracy (Acc), MCC, F1-score (F1), and area under the ROC curve (AUC). These metrics were calculated as follows:

Rec=TPTP+FN

$$Spe=\frac{TN}{TN+FP}$$

$$\mathrm{Pr}e=\frac{TP}{TP+FP}$$

$$Acc=\frac{TP+TN}{TP+TN+FP+FN}$$

$$MCC=\frac{TP.TN-FP.FN}{\sqrt{\left(TP+FP\right)\left(TP+FN\right)\left(TN+FP\right)\left(TN+FN\right)}}$$

F1=2×Rec.PreRec+Pre (13)

where *TP* is True Positive, *TN* is True Negative, *FP* is False Positive, and *FN* is False Negative.

The AUC metric is obtained by plotting recall against (1—specificity) for every threshold and then calculating the area under the curve.

## 3 Results

### 3.1 Models Based on Sequence-Based Feature Set

We calculated the cross-validation performance for each sequence-based feature set using five supervised classifiers: AdaBoost, MLP, RF, SVM, and XGBoost. The performances of these classifiers are shown in Table 4. It can be observed that no classifier is the best for all feature groups. For example, using AAC features, MLP performs the best based on the AUC score. However, using EAAC features, the RF model has the best performance, whereas MLP has the poorest. Among the six different feature sets, the best model achieved was using EAAC features combined with RF, with an AUC score of 0.6999. This model also had the best specificity, precision, and accuracy compared to the other models.

**TABLE 4:** Cross validation result of models from sequence-based features.

Feature groups	Classifier	Rec	Spe	Pre	Acc	MCC	F1	AUC
AAC	Adaboost	0.6120	0.6013	0.2654	0.6033	0.1690	0.3700	0.6433
	MLP	0.6520	0.6192	0.2864	0.6255	0.2150	0.3977	0.6864
	Random Forest	0.6190	0.5809	0.2575	0.5881	0.1576	0.3635	0.6378
	SVM	0.6395	0.5969	0.2714	0.6050	0.1868	0.3808	0.6651
	XGBoost	0.5917	0.5482	0.2353	0.5565	0.1102	0.3362	0.6101
EAAC	Adaboost	0.5983	0.6015	0.2608	0.6009	0.1584	0.3629	0.6384
	MLP	0.5850	0.5946	0.2530	0.5928	0.1422	0.3529	0.6323
	Random Forest	0.6450	0.6598	0.3089	0.6570	0.2450	0.4171	0.6999
	SVM	0.5967	0.6434	0.2821	0.6345	0.1923	0.3827	0.6571
	XGBoost	0.6408	0.6385	0.2945	0.6389	0.2230	0.4030	0.6834
CTDC	Adaboost	0.7050	0.5518	0.2699	0.5809	0.2019	0.3901	0.6641
	MLP	0.6867	0.6034	0.2905	0.6193	0.2300	0.4073	0.6912
	Random Forest	0.6408	0.5676	0.2579	0.5815	0.1639	0.3676	0.6556
	SVM	0.6842	0.5657	0.2705	0.5882	0.1966	0.3874	0.6765
	XGBoost	0.6367	0.5754	0.2605	0.5871	0.1672	0.3693	0.6450
CTDT	Adaboost	0.6208	0.5762	0.2566	0.5847	0.1556	0.3627	0.6261
	MLP	0.6408	0.5756	0.2622	0.5880	0.1708	0.3717	0.6439
	Random Forest	0.6025	0.5982	0.2603	0.5990	0.1588	0.3633	0.6241
	SVM	0.6425	0.5841	0.2661	0.5952	0.1787	0.3760	0.6493
	XGBoost	0.5783	0.5668	0.2390	0.5690	0.1147	0.3378	0.6015
CTDD	Adaboost	0.6358	0.6046	0.2744	0.6106	0.1904	0.3831	0.6531
	MLP	0.5942	0.5365	0.2434	0.5475	0.1120	0.3297	0.6065
	Random Forest	0.6967	0.6164	0.2994	0.6316	0.2476	0.4185	0.6987
	SVM	0.6675	0.6111	0.2877	0.6218	0.2206	0.4017	0.6794
	XGBoost	0.6675	0.6201	0.2927	0.6291	0.2282	0.4064	0.6847
PAAC	Adaboost	0.5942	0.6052	0.2611	0.6031	0.1581	0.3626	0.6253
	MLP	0.5958	0.5717	0.2462	0.5763	0.1321	0.3482	0.6261
	Random Forest	0.6375	0.5809	0.2633	0.5917	0.1723	0.3723	0.6413
	SVM	0.6617	0.5905	0.2752	0.6041	0.1990	0.3885	0.6745
	XGBoost	0.6217	0.5731	0.2554	0.5823	0.1537	0.3615	0.6375
APAAC	Adaboost	0.6125	0.5976	0.2634	0.6004	0.1662	0.3682	0.6367
	MLP	0.5658	0.5904	0.2450	0.5857	0.1237	0.3416	0.6162
	Random Forest	0.6458	0.5831	0.2671	0.5950	0.1805	0.3776	0.6464
	SVM	0.6650	0.5970	0.2794	0.6099	0.2069	0.3932	0.6777
	XGBoost	0.6425	0.5694	0.2596	0.5833	0.1668	0.3695	0.6375

### 3.2 Models Based on Embeddings From Pre-trained Transformer Models

Based on the embeddings extracted from the pre-trained transformer models, we evaluated the same five supervised classifiers. The performance results of the models are presented in Table 5. The combination of the ProtBERT model and SVM can match the recall score with the classic sequence-based feature result. However, all other metrics were lower. In this experiment, the best model with respect to the AUC score was a combination of features from the ProtAlbert model and SVM classifier (AUC = 0.6744). This model also had the highest cross-validation scores for precision, MCC, and F1-score. It can also be noted that out of the six models, SVM performed best on four of them compared to the other machine learning algorithms.

**TABLE 5:** Cross validation result of models from pre-trained transformer models.

Feature groups	Classifier	Rec	Spe	Pre	Acc	MCC	F1	AUC
ProtBERT	Adaboost	0.5767	0.5680	0.2389	0.5697	0.1142	0.3374	0.5996
	MLP	0.5892	0.5608	0.2395	0.5662	0.1187	0.3396	0.6128
	Random Forest	0.5567	0.6426	0.2681	0.6262	0.1602	0.3616	0.6415
	SVM	0.7042	0.4775	0.2420	0.5207	0.1475	0.3578	0.6275
	XGBoost	0.6033	0.6007	0.2619	0.6012	0.1616	0.3649	0.6398
ProtBert-BFD	Adaboost	0.5433	0.5547	0.2231	0.5525	0.0773	0.3162	0.5776
	MLP	0.5900	0.5645	0.2420	0.5694	0.1218	0.3430	0.6076
	Random Forest	0.5383	0.6230	0.2510	0.6069	0.1289	0.3421	0.6122
	SVM	0.6242	0.5819	0.2595	0.5899	0.1626	0.3662	0.6420
	XGBoost	0.5908	0.5733	0.2453	0.5766	0.1295	0.3464	0.6142
ProtAlbert	Adaboost	0.5875	0.5753	0.2450	0.5776	0.1284	0.3456	0.6193
	MLP	0.5858	0.6189	0.2657	0.6126	0.1646	0.3615	0.6407
	Random Forest	0.5808	0.6316	0.2703	0.6220	0.1697	0.3687	0.6535
	SVM	0.6283	0.6136	0.2767	0.6164	0.1919	0.3840	0.6744
	XGBoost	0.6092	0.5927	0.2604	0.5958	0.1597	0.3646	0.6477
ProtT5-XL-UniRef50	Adaboost	0.5533	0.5655	0.2306	0.5632	0.0938	0.3254	0.5897
	MLP	0.6192	0.5633	0.2501	0.5739	0.1439	0.3558	0.6296
	Random Forest	0.5608	0.6171	0.2562	0.6064	0.1419	0.3515	0.6237
	SVM	0.6583	0.5710	0.2653	0.5876	0.1807	0.3777	0.6600
	XGBoost	0.5933	0.5807	0.2497	0.5831	0.1377	0.3509	0.6183
ProtT5-XL-BFD	Adaboost	0.5892	0.5600	0.2395	0.5656	0.1175	0.3405	0.5959
	MLP	0.6000	0.5768	0.2502	0.5812	0.1396	0.3529	0.6188
	Random Forest	0.5392	0.6163	0.2485	0.6017	0.1242	0.3399	0.6145
	SVM	0.6550	0.5625	0.2604	0.5801	0.1711	0.3724	0.6548
	XGBoost	0.5858	0.5862	0.2490	0.5862	0.1361	0.3489	0.6224
ProtXLNet	Adaboost	0.5125	0.5343	0.2057	0.5302	0.0369	0.2934	0.5421
	MLP	0.5325	0.5248	0.2081	0.5262	0.0450	0.2991	0.5463
	Random Forest	0.5050	0.5668	0.2152	0.5551	0.0568	0.3015	0.5511
	SVM	0.4742	0.5770	0.2103	0.5575	0.0408	0.2900	0.5460
	XGBoost	0.5642	0.5504	0.2274	0.5530	0.0902	0.3238	0.5652

### 3.3 Models Based on Combination of Sequence-Based Feature and Pre-trained Transformer Models Feature Set

To obtain the best model, we tested various combinations of two or more feature sets to evaluate further models, such as AAC-EAAC, AAC-CTDC, and AAC-ProtBert. For this, we limited the embedding features to a maximum of one set in the combination. Similar to previous experiments, five classification algorithms were used: AdaBoost, XGBoost, SVM (RBF kernel), RF, and MLP.

**FIGURE 2:**
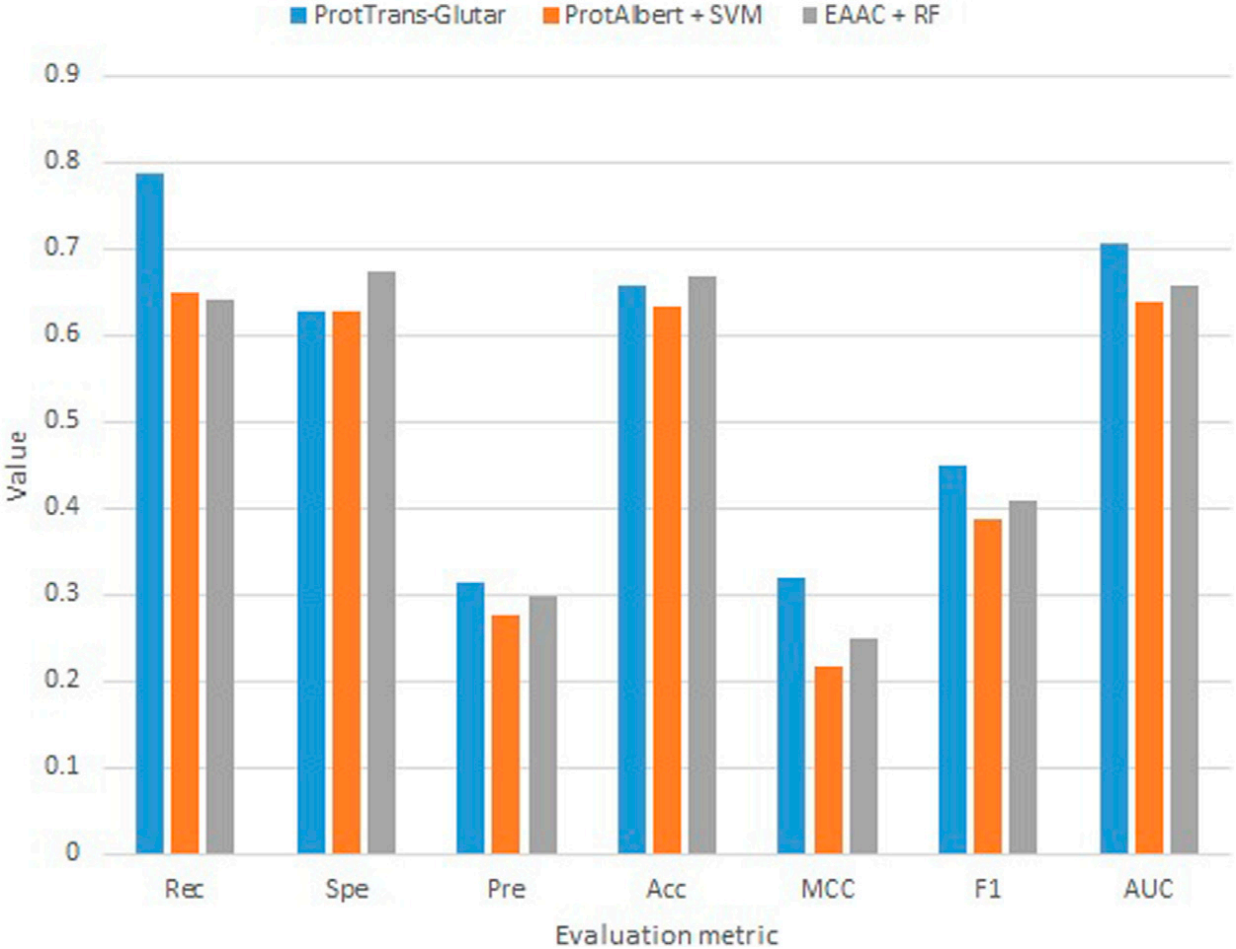
Independent test evaluation of the best models from each group.

Our best model, ProtTrans-Glutar, uses a combination of the features CTDD, EAAC, and ProtT5-XL-UniRef50 with the XGBoost classification algorithm. The performance of this model is shown in Table 6, with comparison to the best model from sequence-based features (EAAC with RF classifier) and the best model from embeddings of the protein model (ProtAlbert with SVM classifier). According to the cross-validation performance on training data, this model has the best AUC and recall compared with models with features from only one group. These three models were then evaluated using an independent dataset (Figure 2). This test result shows that ProtTrans-Glutar outperformed the other two models in terms of AUC, recall, precision, MCC, and F1-score. However, it is severely worse in terms of specificity and slightly worse in terms of accuracy compared to the EAAC + RF model.

**TABLE 6:** Performance comparison of the best models in each group.

Evaluation	Models	Length	Rec	Spe	Pre	Acc	MCC	F1	AUC
10-fold CV on Training Data	ProtTrans-Glutar^a^	1,599	0.6783	0.6277	0.3004	0.6374	0.2433	0.4158	0.7093
	ProtAlbert + SVM	4,096	0.6283	0.6136	0.2767	0.6164	0.1919	0.3840	0.6744
	EAAC + RF	380	0.6450	0.6598	0.3089	0.6570	0.2450	0.4171	0.6999
Independent Test Set	ProtTrans-Glutar^a^	1,599	0.7864	0.6286	0.3147	0.6567	0.3196	0.4494	0.7075
	ProtAlbert + SVM	4,096	0.6500	0.6286	0.2753	0.6324	0.2161	0.3866	0.6393
	EAAC + RF	380	0.6409	0.6739	0.2989	0.6680	0.2479	0.4076	0.6574

a Model uses combined features CTDD-EAAC-ProtT5XLUniRef50 with XGBoost classifier.

**FIGURE 3:**
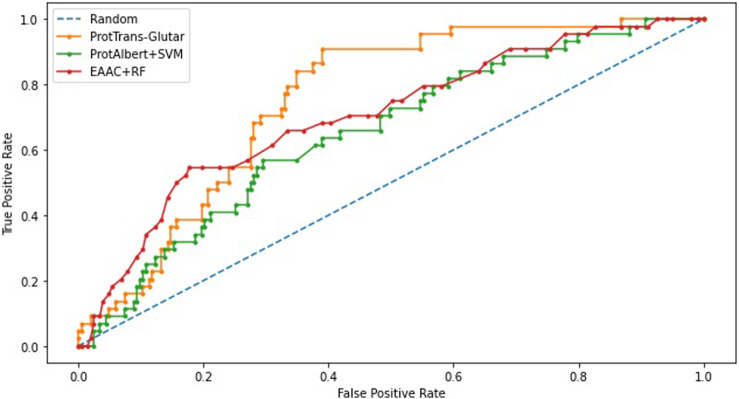
ROC-Curve plot of best models in each group.

As shown in the ROC curves of the three models (Figure 3), EAAC + RF performed better for low values of FPR, but for larger values, ProtTrans-Glutar performed better. It is also noted that ProtAlbert + SVM performed worse for most values of FPR. Overall, ProtTrans-Glutar was the best model with an AUC of 0.7075.

## 4 Discussion

From our study, it was shown that building prediction models from traditional sequence-based features only provided limited performance (Table 4). It was also shown that using only embeddings from pre-trained protein models gave slightly worse results, except that the recall performance was almost the same (Table 5). When we combined the features from these two groups, we found that the best performance was achieved by the combination of the features CTDD, EAAC, and ProtT5-XL-UniRef50 with the XGBoost classifier (independent test AUC = 0.7075). This indicated that ProtT5-XL-UniRef50 features on their own are not the best embedding model during the individual feature evaluation (see Table 5), but combined with CTDD and EAAC, it outperformed the other models. It is worth mentioning that Elnaggar et al. (2021), who developed and trained protein models, revealed that ProtT5 models outperformed state-of-the-art models in protein classification tasks, namely in prediction of localization (10-class classification) and prediction of membrane/other (binary classification), compared to other embedding models.

For further evaluation, we compared our model with previous glutarylation site prediction models (Table 7). The first three models, GlutPred, iGlu-Lys, and MDDGlutar, used datasets that were different from our model and are shown for reference. The other model, iGlu_Adaboost, utilized the same public dataset as for our model and contained glutarylation sites from the same four species. ProtTrans-Glutar outperformed the other models in terms of the recall performance (Rec = 0.7864 for unbalanced data). This high recall suggests that this model can be useful for uncovering new and potential glutarylation sites.

**TABLE 7:** Performance comparison of existing models.

Models	Resources	Rec	Spe	Pre	Acc	MCC	F1	AUC
GlutPred	PLMD	0.5179	0.7850	0.2397	0.7541	0.2238	n/a	0.7663
iGlu-Lys	PLMD	0.5143	0.9531	n/a	0.8853	0.52	n/a	0.8842
MDDGlutar	PLMD	0.652	0.739	n/a	0.71	0.38	n/a	n/a
iGlu_AdaBoost	PLMD, NCBI, Swiss-Prot	0.7273	0.7192	0.3596	0.7207	0.36	0.48	0.6300
ProtTrans-Glutar	PLMD, NCBI, Swiss-Prot	0.7822	0.6286	0.3147	0.6567	0.3196	0.4494	0.7075

Furthermore, we also evaluated our model by using a balanced training and testing dataset using random under-sampling for comparison with the RF-GlutarySite model (Table 8), which uses the same dataset but is balanced before evaluating performance. Because the authors of RF-GlutarySite did not provide their data after the resampling process, we performed the experiments 10 times to handle variance from the under-sampling. The ProtTrans-Glutar model showed a higher recall score of 0.7864 compared to RF-GlutarySite (0.7410), in addition to a slightly higher accuracy, MCC, and F1-score. However, the specificity and precision scores were lower.

**TABLE 8:** Performance comparison with RF-GlutarySite using balanced train and test data.

Models	Resources	Rec	Spe	Pre	Acc	MCC	F1	AUC
RF-GlutarySite^a^	PLMD, NCBI, Swiss-Prot	0.741	0.685	0.72	0.713	0.43	0.72	0.72
ProtTrans-Glutar (balanced)	PLMD, NCBI, Swiss-Prot	0.7864	0.6455	0.6955	0.7159	0.4388	0.7358	0.7159

a RF-GlutarySite model balanced the training and testing dataset using undersampling.

In summary, the model improved the recall score compared to the existing models but did not improve other metrics. However, we would like to point out that GlutPred, iGlu-Lys, and MDDGlutar based their glutarylation datasets on less diverse sources (two species only), whereas ProtTrans-Glutar with RF-GlutarySite and iGlu_Adaboost utilized newer datasets (four species). The more diverse source of glutarylation sites in the data may present more difficulty in improving performance, especially in terms of specificity and accuracy. Compared with iGlu_Adaboost, which used the same dataset, our model improved their recall and AUC scores. Despite this, the specificity is worse and will be a challenge for future research.

## 5 Summary

In this study, we presented a new glutarylation site predictor by incorporating embeddings from pretrained protein models as features. This method, which is termed ProtTrans-Glutar, combines three feature sets: EAAC, CTDD, and ProtT5-XL-UniRef50. Random under-sampling was used in conjunction with the XGBoost classifier to train the model. The performance evaluations obtained from this model for recall, specificity, and AUC are 0.7864, 0.6286, and 0.7075, respectively. Compared to other models using the same dataset of more diverse sources of glutarylation sites, this model outperformed the existing model in terms of recall and AUC score and could potentially be used to complement previous models to reveal new glutarylated sites. In the future, refinements can be expected through further experiments, such as applying other feature selection methods, feature processing, and investigating deep learning models.

## Data Availability

Publicly available datasets were analyzed in this study. This data can be found here: https://github.com/findriani/ProtTrans-Glutar/tree/main/dataset.
