# Supplementary material for: ProtTrans-Glutar: Incorporating Features From Pre-trained Transformer-Based Models for Predicting Glutarylation Sites
Source: Front Genet. 2022 May 31;13:885929. doi: 10.3389/fgene.2022.885929 (PMC9194472; doi:10.3389/fgene.2022.885929)
Supplement: Supplementary file 1 [file DataSheet1.docx]

Supplementary Material

**Supplementary File S1**

In this supplementary material, the 7 feature extraction methods are explained in more details. The protein sequence “IEIPEVRLNLEKMMEQKHSAVKA” is used as an example in all the methods. These methods are part of

1. Amino Acid Composition (AAC)

The protein sequence is converted into a vector of length 20, representing the frequency of the 20 amino acids (“*ACDEFGHIKLMNPQRSTVWY*”). Each element is calculated according to Equation 1, as follows:

$f\left( t \right)=\frac{N\left( t \right)}{N}$ (1)

where *t* is the amino acid type, *N(t)* is the total number of amino acids *t* appearing in the sequence, and *N* is the length of the sequence.

Example: IEIPEVRLNLEKMMEQKHSAVKA

The frequency of amino acid A is 2 / 23 = 0.08695652174

The frequency of amino acid B is 0 / 23 = 0

The frequency of amino acid C is 0 / 23 = 0

And so on.

The whole AAC features extracted are as follows:

| AAC.A | 0.086957 |
| --- | --- |
| AAC.C | 0 |
| AAC.D | 0 |
| AAC.E | 0.173913 |
| AAC.F | 0 |
| AAC.G | 0 |
| AAC.H | 0.043478 |
| AAC.I | 0.086957 |
| AAC.K | 0.130435 |
| AAC.L | 0.086957 |
| AAC.M | 0.086957 |
| AAC.N | 0.043478 |
| AAC.P | 0.043478 |
| AAC.Q | 0.043478 |
| AAC.R | 0.043478 |
| AAC.S | 0.043478 |
| AAC.T | 0 |
| AAC.V | 0.086957 |
| AAC.W | 0 |
| AAC.Y | 0 |

1. Enhanced Amino Acid Composition (EAAC)

In this encoding, the EAAC is calculated using sliding windows, that is, from a fixed window size, moving from left to right. To calculate the frequency of each amino acid in each window, see Equation 2:

- $f\left( t,win \right)=\frac{N\left( t,win \right)}{N\left( win \right)}$ (2)

where *N(t,win)* represents the number of amino acids *t* that appear in the window *win* and *N(win)* represents the length of the window.

Example: IEIPEVRLNLEKMMEQKHSAVKA

With window length 5, the sub-sequence of the windows are IEIPE, EIPEV, IPEVR, …, SAVKA

Window 1: IEIPE

The amino acid composition of Window 1

| EAAC.SW.1.A | 0 |
| --- | --- |
| EAAC.SW.1.C | 0 |
| EAAC.SW.1.D | 0 |
| EAAC.SW.1.E | 0.4 |
| EAAC.SW.1.F | 0 |
| EAAC.SW.1.G | 0 |
| EAAC.SW.1.H | 0 |
| EAAC.SW.1.I | 0.4 |
| EAAC.SW.1.K | 0 |
| EAAC.SW.1.L | 0 |
| EAAC.SW.1.M | 0 |
| EAAC.SW.1.N | 0 |
| EAAC.SW.1.P | 0.2 |
| EAAC.SW.1.Q | 0 |
| EAAC.SW.1.R | 0 |
| EAAC.SW.1.S | 0 |
| EAAC.SW.1.T | 0 |
| EAAC.SW.1.V | 0 |
| EAAC.SW.1.W | 0 |
| EAAC.SW.1.Y | 0 |

Window 2: EIPEV

Amino acid composition of Window 2

| EAAC.SW.2.A | 0 |
| --- | --- |
| EAAC.SW.2.C | 0 |
| EAAC.SW.2.D | 0 |
| EAAC.SW.2.E | 0.4 |
| EAAC.SW.2.F | 0 |
| EAAC.SW.2.G | 0 |
| EAAC.SW.2.H | 0 |
| EAAC.SW.2.I | 0.2 |
| EAAC.SW.2.K | 0 |
| EAAC.SW.2.L | 0 |
| EAAC.SW.2.M | 0 |
| EAAC.SW.2.N | 0 |
| EAAC.SW.2.P | 0.2 |
| EAAC.SW.2.Q | 0 |
| EAAC.SW.2.R | 0 |
| EAAC.SW.2.S | 0 |
| EAAC.SW.2.T | 0 |
| EAAC.SW.2.V | 0.2 |
| EAAC.SW.2.W | 0 |
| EAAC.SW.2.Y | 0 |

…

Window 19: SAVKA

Amino acid composition of Window 19

| EAAC.SW.19.A | 0.4 |
| --- | --- |
| EAAC.SW.19.C | 0 |
| EAAC.SW.19.D | 0 |
| EAAC.SW.19.E | 0 |
| EAAC.SW.19.F | 0 |
| EAAC.SW.19.G | 0 |
| EAAC.SW.19.H | 0 |
| EAAC.SW.19.I | 0 |
| EAAC.SW.19.K | 0.2 |
| EAAC.SW.19.L | 0 |
| EAAC.SW.19.M | 0 |
| EAAC.SW.19.N | 0 |
| EAAC.SW.19.P | 0 |
| EAAC.SW.19.Q | 0 |
| EAAC.SW.19.R | 0 |
| EAAC.SW.19.S | 0.2 |
| EAAC.SW.19.T | 0 |
| EAAC.SW.19.V | 0.2 |
| EAAC.SW.19.W | 0 |
| EAAC.SW.19.Y | 0 |

Combined, all amino acid composition from 19 windows has a total length of 380.

1. Composition (CTDC)

Thirteen properties are used to build the features for CTDC, CTDT, and CTDD. Each property is divided into three groups (see Table S1). For example, the attribute “Hydrophobicity_PRAM900101” divides the amino acids into polar, neutral, and hydrophobic groups.

Table S1. Physicochemical attributes and its division of the amino acids

| Attribute | Division | | |
| --- | --- | --- | --- |
| Hydrophobicity_PRAM900101 | Polar: RKEDQN | Neutral: GASTPHY | Hydrophobicity: CLVIMFW |
| Hydrophobicity_ARGP820101 | Polar: QSTNGDE | Neutral: RAHCKMV | Hydrophobicity: LYPFIW |
| Hydrophobicity_ZIMJ680101 | Polar: QNGSWTDERA | Neutral: HMCKV | Hydrophobicity: LPFYI |
| Hydrophobicity_PONP930101 | Polar: KPDESNQT | Neutral: GRHA | Hydrophobicity: YMFWLCVI |
| Hydrophobicity_CASG920101 | Polar: KDEQPSRNTG | Neutral: AHYMLV | Hydrophobicity: FIWC |
| Hydrophobicity_ENGD860101 | Polar: RDKENQHYP | Neutral :SGTAW | Hydrophobicity: CVLIMF |
| Hydrophobicity_FASG890101 | Polar: KERSQD | Neutral: NTPG | Hydrophobicity: AYHWVMFLIC |
| Normalized van der Waals volume | Volume range: 0-2.78  GASTPD | Volume range: 2.95-94.0  NVEQIL | Volume range: 4.03-8.08  MHKFRYW |
| Polarity | Polarity value: 4.9-6.2  LIFWCMVY | Polarity value: 8.0-9.2  PATGS | Polarity value: 10.4-13.0  HQRKNED |
| Polarizability | Polarizability value: 0-1.08  GASDT | Polarizability value: 0.128-120.186  GPNVEQIL | Polarizability value: 0.219-0.409  KMHFRYW |
| Charge | Positive: KR | Neutral: ANCQGHILMFPSTWYV | Negative: DE |
| Secondary structure | Helix: EALMQKRH | Strand: VIYCWFT | Coil: GNPSD |
| Solvent accessibility | Buried: ALFCGIVW | Exposed: PKQEND | Intermediate: MPSTHY |

For Composition (CTDC), an attribute contributes to three values, representing the global distribution (frequency) of the amino acids in each of the three groups of attributes. The composition is computed as follows:

$C\left( r \right)=\frac{N\left( r \right)}{N}$ (3)

where *N(r)* is the number of occurrences of type *r* amino acids in the sequence and *N* is the length of the sequence.

Example: IEIPEVRLNLEKMMEQKHSAVKA

**Attribute *Hydrophobicity_PRAM900101***

Polar: RKEDQN

Neutral: GASTPHY

Hydrophobicity: CLVIMFW

Calculate the composition of group Polar.

$$C\left( Polar \right)$$

$$=\frac{count\left( R \right)+count\left( K \right)+count\left( E \right)+count\left( D \right)+count\left( Q \right)+count\left( N \right)}{23}$$

$$=\frac{1+3+4+0+1+1}{23}=\frac{10}{23}=0.434783$$

The other 2 groups Neutral and Hydrophobicity are calculated similarly.

C(Neutral) = 0.217391304

C(Hydrophobicity) = 0.347826087

**Attribute *Hydrophobicity_ARGP820101***

Polar: QSTNGDE

Neutral: RAHCKMV

Hydrophobicity: LYPFIW

As above, calculate the Composition of each group.

C(Polar) = 0.304347826

C(Neutral) = 0.47826087

C(Hydrophobicity) = 0.217391304

Each attribute contributes to 3 composition values, one from each group. Combined, all 13 attributes will give 39 values as the feature set.

| CTDC.hydrophobicity_PRAM900101.G1 | 0.434783 |
| --- | --- |
| CTDC.hydrophobicity_PRAM900101.G2 | 0.217391 |
| CTDC.hydrophobicity_PRAM900101.G3 | 0.347826 |
| CTDC.hydrophobicity_ARGP820101.G1 | 0.304348 |
| CTDC.hydrophobicity_ARGP820101.G2 | 0.478261 |
| CTDC.hydrophobicity_ARGP820101.G3 | 0.217391 |
| CTDC.hydrophobicity_ZIMJ680101.G1 | 0.434783 |
| CTDC.hydrophobicity_ZIMJ680101.G2 | 0.347826 |
| CTDC.hydrophobicity_ZIMJ680101.G3 | 0.217391 |
| CTDC.hydrophobicity_PONP930101.G1 | 0.478261 |
| CTDC.hydrophobicity_PONP930101.G2 | 0.173913 |
| CTDC.hydrophobicity_PONP930101.G3 | 0.347826 |
| CTDC.hydrophobicity_CASG920101.G1 | 0.521739 |
| CTDC.hydrophobicity_CASG920101.G2 | 0.391304 |
| CTDC.hydrophobicity_CASG920101.G3 | 0.086957 |
| CTDC.hydrophobicity_ENGD860101.G1 | 0.521739 |
| CTDC.hydrophobicity_ENGD860101.G2 | 0.130435 |
| CTDC.hydrophobicity_ENGD860101.G3 | 0.347826 |
| CTDC.hydrophobicity_FASG890101.G1 | 0.434783 |
| CTDC.hydrophobicity_FASG890101.G2 | 0.086957 |
| CTDC.hydrophobicity_FASG890101.G3 | 0.478261 |
| CTDC.normwaalsvolume.G1 | 0.173913 |
| CTDC.normwaalsvolume.G2 | 0.521739 |
| CTDC.normwaalsvolume.G3 | 0.304348 |
| CTDC.polarity.G1 | 0.347826 |
| CTDC.polarity.G2 | 0.173913 |
| CTDC.polarity.G3 | 0.478261 |
| CTDC.polarizability.G1 | 0.130435 |
| CTDC.polarizability.G2 | 0.565217 |
| CTDC.polarizability.G3 | 0.304348 |
| CTDC.charge.G1 | 0.173913 |
| CTDC.charge.G2 | 0.652174 |
| CTDC.charge.G3 | 0.173913 |
| CTDC.secondarystruct.G1 | 0.695652 |
| CTDC.secondarystruct.G2 | 0.173913 |
| CTDC.secondarystruct.G3 | 0.130435 |
| CTDC.solventaccess.G1 | 0.347826 |
| CTDC.solventaccess.G2 | 0.434783 |
| CTDC.solventaccess.G3 | 0.217391 |

1. Transition (CTDT)

For transition (CTDT), an attribute contributes to three values, each representing the number of transitions between any pair of groups. The transition is calculated as follows:

$T\left( r,s \right)=\frac{N\left( r,s \right)+N\left( s,r \right)}{N-1}$ (4)

where *N(r,s)* represents the number of occurrences amino acid type *r* transit to type *s* (i.e it appeared as “rs” in the sequence), and *N* is the length of the sequence. Similarly, N(s,r) is the reverse, that is, the number of “sr” occurrences in the sequence.

Example: IEIPEVRLNLEKMMEQKHSAVKA

**Attribute *Hydrophobicity_PRAM900101***

Polar: RKEDQN

Neutral: GASTPHY

Hydrophobicity: CLVIMFW

Consider each transition in the sequence, I to E, E to I, I to P, …, K to A

Transition I to E, is a Polar-Hydrophobicity transition.

Transition E to I, is a Polar-Hydrophobicity transition

Transition I to P, is Neutral-Hydrophobicity transition

…

Transition K to A, is a Polar-Neutral transition

The frequency of each transition type is as follows:

T(Polar,Neutral) = 0.136363636

T(Polar,Hydrophobicity) = 0.5

T(Neutral,Hydrophobicity)=0.090909091

Transition frequency is similarly calculated on other attributes. Each attribute contributes to 3 Transition values, one for each pair of group. Combined, all 13 attributes will give 39 values as the feature set

| CTDT.hydrophobicity_PRAM900101.Tr1221 | 0.136364 |
| --- | --- |
| CTDT.hydrophobicity_PRAM900101.Tr1331 | 0.5 |
| CTDT.hydrophobicity_PRAM900101.Tr2332 | 0.090909 |
| CTDT.hydrophobicity_ARGP820101.Tr1221 | 0.272727 |
| CTDT.hydrophobicity_ARGP820101.Tr1331 | 0.272727 |
| CTDT.hydrophobicity_ARGP820101.Tr2332 | 0.045455 |
| CTDT.hydrophobicity_ZIMJ680101.Tr1221 | 0.363636 |
| CTDT.hydrophobicity_ZIMJ680101.Tr1331 | 0.318182 |
| CTDT.hydrophobicity_ZIMJ680101.Tr2332 | 0 |
| CTDT.hydrophobicity_PONP930101.Tr1221 | 0.181818 |
| CTDT.hydrophobicity_PONP930101.Tr1331 | 0.454545 |
| CTDT.hydrophobicity_PONP930101.Tr2332 | 0.136364 |
| CTDT.hydrophobicity_CASG920101.Tr1221 | 0.590909 |
| CTDT.hydrophobicity_CASG920101.Tr1331 | 0.136364 |
| CTDT.hydrophobicity_CASG920101.Tr2332 | 0 |
| CTDT.hydrophobicity_ENGD860101.Tr1221 | 0.090909 |
| CTDT.hydrophobicity_ENGD860101.Tr1331 | 0.545455 |
| CTDT.hydrophobicity_ENGD860101.Tr2332 | 0.045455 |
| CTDT.hydrophobicity_FASG890101.Tr1221 | 0.045455 |
| CTDT.hydrophobicity_FASG890101.Tr1331 | 0.590909 |
| CTDT.hydrophobicity_FASG890101.Tr2332 | 0.136364 |
| CTDT.normwaalsvolume.Tr1221 | 0.136364 |
| CTDT.normwaalsvolume.Tr1331 | 0.090909 |
| CTDT.normwaalsvolume.Tr2332 | 0.272727 |
| CTDT.polarity.Tr1221 | 0.090909 |
| CTDT.polarity.Tr1331 | 0.5 |
| CTDT.polarity.Tr2332 | 0.136364 |
| CTDT.polarizability.Tr1221 | 0.045455 |
| CTDT.polarizability.Tr1331 | 0.090909 |
| CTDT.polarizability.Tr2332 | 0.272727 |
| CTDT.charge.Tr1221 | 0.318182 |
| CTDT.charge.Tr1331 | 0.045455 |
| CTDT.charge.Tr2332 | 0.318182 |
| CTDT.secondarystruct.Tr1221 | 0.272727 |
| CTDT.secondarystruct.Tr1331 | 0.227273 |
| CTDT.secondarystruct.Tr2332 | 0.045455 |
| CTDT.solventaccess.Tr1221 | 0.454545 |
| CTDT.solventaccess.Tr1331 | 0.090909 |
| CTDT.solventaccess.Tr2332 | 0.181818 |

1. Distribution (CTDD)

The distribution feature consists of five values per attribute group, each of which corresponds to the fraction of the sequence length at five different positions in the group: first occurrence, 25%, 50%, 75%, and 100%

Example: IEIPEVRLNLEKMMEQKHSAVKA

**Attribute *Hydrophobicity_PRAM900101***

Polar: RKEDQN

Neutral: GASTPHY

Hydrophobicity: CLVIMFW

Polar-First occurrence = 2/23 = 8.695652174%

Polar-25% = 5/23 = 21.73913043

Polar-50% = 11/23 = 47.82608696

Polar-75% = 15/23 = 65.2173913

Polar-100% = 22/23 = 95.65217391

Distribution descriptor is similarly calculated on other attributes. Each attribute contributes to 5 Distribution values. Combined, all 13 attributes will give 65 values as the feature set

1. PAAC (Pseudo Amino Acid Composition)

For protein sequence P with L amino acid residues P = [R_1_R_2_R_3_…R_L_], the PAAC features can be formulated as P= [p_1_, p_2_, …, p_20_, p_20+1_, …, p_20+λ_]^T^ , (λ< L)

$$p_{u}=\left\{ \begin{aligned} \frac{f_{u}}{\sum_{i=1}^{20} f_{i}+w\sum_{k=1}^{\lambda} \tau_{k}},\left( 1\leq u\leq20 \right) \\ \frac{{w\tau}_{u-20}}{\sum_{i=1}^{20} f_{i}+w\sum_{k=1}^{\lambda} \tau_{k}},\left( 20+1\leq u\leq20+\lambda\right) \end{aligned} \right.$$

Where w is the weight factor and $\tau_{k}$ is the k-the tier correlation factor, defined as

$$\tau_{k}=\frac{1}{L-k}\sum_{i=1}^{L-k} J_{i,i+k}, \left( k<L \right)$$

and

$$J_{i,i+k}=\frac{1}{\Gamma}\sum_{q=1}^{\Gamma} \left[ \Phi_{q}R_{i+k}-\Phi_{q}R_{i} \right]^{2}$$

Where Ф*_q_*(*R_i_*) is the *q*-th function of the amino acid *R_i_*, and Г the total number of functions. In here Г=3 and the functions used are hydrophobicity value, hydrophilicity value, and side chain mass of amino acid *R_i_*

Example: IEIPEVRLNLEKMMEQKHSAVKA

| PAAC.Xc1.A | 0.823334 |
| --- | --- |
| PAAC.Xc1.R | 0.411667 |
| PAAC.Xc1.N | 0.411667 |
| PAAC.Xc1.D | 0 |
| PAAC.Xc1.C | 0 |
| PAAC.Xc1.Q | 0.411667 |
| PAAC.Xc1.E | 1.646668 |
| PAAC.Xc1.G | 0 |
| PAAC.Xc1.H | 0.411667 |
| PAAC.Xc1.I | 0.823334 |
| PAAC.Xc1.L | 0.823334 |
| PAAC.Xc1.K | 1.235001 |
| PAAC.Xc1.M | 0.823334 |
| PAAC.Xc1.F | 0 |
| PAAC.Xc1.P | 0.411667 |
| PAAC.Xc1.S | 0.411667 |
| PAAC.Xc1.T | 0 |
| PAAC.Xc1.W | 0 |
| PAAC.Xc1.Y | 0 |
| PAAC.Xc1.V | 0.823334 |
| PAAC.Xc2.lambda1 | 0.051346 |
| PAAC.Xc2.lambda2 | 0.033433 |
| PAAC.Xc2.lambda3 | 0.040146 |
| PAAC.Xc2.lambda4 | 0.042338 |
| PAAC.Xc2.lambda5 | 0.026775 |
| PAAC.Xc2.lambda6 | 0.048352 |
| PAAC.Xc2.lambda7 | 0.030635 |
| PAAC.Xc2.lambda8 | 0.043705 |
| PAAC.Xc2.lambda9 | 0.047359 |
| PAAC.Xc2.lambda10 | 0.023432 |
| PAAC.Xc2.lambda11 | 0.042022 |
| PAAC.Xc2.lambda12 | 0.043776 |
| PAAC.Xc2.lambda13 | 0.033219 |
| PAAC.Xc2.lambda14 | 0.059726 |
| PAAC.Xc2.lambda15 | 0.022069 |

1. APAAC

For a protein sample P with L amino acid residues P = [R_1_R_2_R_3_…R_L_], APAAC features is formulated as

P= [p_1_, p_2_, …, p_20_, p_20+1_, …, p_20+λ_, p_20+λ, …_, p_2λ_]^T^ , (λ< L)

where

$p_{u}=\left\{ \begin{aligned} \frac{f_{u}}{\sum_{i=1}^{20} f_{i}+w\sum_{j=1}^{2\lambda} \tau_{k}},\left( 1\leq u\leq20 \right) \\ \frac{{w\tau}_{u-20}}{\sum_{i=1}^{20} f_{i}+w\sum_{j=1}^{2\lambda} \tau_{k}},\left( 20+1\leq u\leq20+2\lambda\right) \end{aligned} \right.$

Example: IEIPEVRLNLEKMMEQKHSAVKA

| APAAC.Pc1.A | 1.867215 |
| --- | --- |
| APAAC.Pc1.R | 0.933608 |
| APAAC.Pc1.N | 0.933608 |
| APAAC.Pc1.D | 0 |
| APAAC.Pc1.C | 0 |
| APAAC.Pc1.Q | 0.933608 |
| APAAC.Pc1.E | 3.734431 |
| APAAC.Pc1.G | 0 |
| APAAC.Pc1.H | 0.933608 |
| APAAC.Pc1.I | 1.867215 |
| APAAC.Pc1.L | 1.867215 |
| APAAC.Pc1.K | 2.800823 |
| APAAC.Pc1.M | 1.867215 |
| APAAC.Pc1.F | 0 |
| APAAC.Pc1.P | 0.933608 |
| APAAC.Pc1.S | 0.933608 |
| APAAC.Pc1.T | 0 |
| APAAC.Pc1.W | 0 |
| APAAC.Pc1.Y | 0 |
| APAAC.Pc1.V | 1.867215 |
| APAAC.Pc2.Hydrophobicity.1 | -0.02223 |
| APAAC.Pc2.Hydrophilicity.1 | -0.01705 |
| APAAC.Pc2.Hydrophobicity.2 | 0.010748 |
| APAAC.Pc2.Hydrophilicity.2 | 0.006387 |
| APAAC.Pc2.Hydrophobicity.3 | -0.00607 |
| APAAC.Pc2.Hydrophilicity.3 | 0.002221 |
| APAAC.Pc2.Hydrophobicity.4 | -0.01014 |
| APAAC.Pc2.Hydrophilicity.4 | 3.61E-05 |
| APAAC.Pc2.Hydrophobicity.5 | 0.025866 |
| APAAC.Pc2.Hydrophilicity.5 | 0.024043 |
| APAAC.Pc2.Hydrophobicity.6 | -0.01742 |
| APAAC.Pc2.Hydrophilicity.6 | -0.00048 |
| APAAC.Pc2.Hydrophobicity.7 | 0.012663 |
| APAAC.Pc2.Hydrophilicity.7 | 0.014859 |
| APAAC.Pc2.Hydrophobicity.8 | -0.00869 |
| APAAC.Pc2.Hydrophilicity.8 | -0.0027 |
| APAAC.Pc2.Hydrophobicity.9 | -0.01129 |
| APAAC.Pc2.Hydrophilicity.9 | -0.01233 |
| APAAC.Pc2.Hydrophobicity.10 | 0.025316 |
| APAAC.Pc2.Hydrophilicity.10 | 0.035179 |
| APAAC.Pc2.Hydrophobicity.11 | -0.00442 |
| APAAC.Pc2.Hydrophilicity.11 | 0.000702 |
| APAAC.Pc2.Hydrophobicity.12 | -0.00811 |
| APAAC.Pc2.Hydrophilicity.12 | -0.00062 |
| APAAC.Pc2.Hydrophobicity.13 | 0.007781 |
| APAAC.Pc2.Hydrophilicity.13 | 0.017787 |
| APAAC.Pc2.Hydrophobicity.14 | -0.03558 |
| APAAC.Pc2.Hydrophilicity.14 | -0.02392 |
| APAAC.Pc2.Hydrophobicity.15 | 0.027795 |
| APAAC.Pc2.Hydrophilicity.15 | 0.036064 |
